# The Response of Grain Yield and Root Morphological and Physiological Traits to Nitrogen Levels in Paddy Rice

**DOI:** 10.3389/fpls.2021.713814

**Published:** 2021-08-31

**Authors:** Wei Xin, Hualong Liu, Hongwei Zhao, Jingguo Wang, Hongliang Zheng, Yan Jia, Luomiao Yang, Xinpeng Wang, Jiaming Li, Xianwei Li, Lei Lei, Detang Zou

**Affiliations:** Key Laboratory of Germplasm Enhancement, Physiology and Ecology of Food Crops in Cold Region, Ministry of Education, Northeast Agricultural University, Harbin, China

**Keywords:** japonica cultivar, nitrogen uptake, grain yield, root morphophysiology, phytohormones

## Abstract

Rice (*Oryza sativa* L.) is an important crop in China. Although it is known that its yield is restricted by nitrogen (N) supply, the response of the root system to N supply specifically has not been systematically explored. This study aimed to investigate the effect of N uptake on grain yield to clarify the relationships between root morphophysiological traits and N uptake, and to understand relation between phytohormones and root morphophysiological traits. Two N-efficient absorption cultivars (NEAs) and two N-inefficient absorption cultivars (NIAs) were grown in the field, and three N conditions, deficient N (60 kg ha^–1^), intermediate N (180 kg ha^–1^), and sufficient N (240 kg ha^–1^), were applied during the growing season. The results showed higher dry matter and grain yield in NEAs than in NIAs, which was mainly attributed to increased N uptake in the mid- and late growth stages under all N conditions. And NEAs have different root regulation methods to obtain higher N accumulation and yield under different N supply conditions. Under lower N conditions, compared with NIAs, NEAs shown greater total root length, root oxidation activity, and root active absorbing surface area and smaller root diameter owing to higher indole-3-acetic acid and cytokinin content and lower 1-aminocyclopropane-1-carboxylic acid content in the early growth stages to respond to low N stress faster, laying a morphophysiological basis for its high N-uptake capacity in the mid- and late growth stages. Under higher N conditions, NEAs had higher root oxidation activity and root active absorbing surface area for N uptake and yield formation owing to higher abscisic acid and cytokinin content in the mid- and late growth stages, which improved the seed setting rate, thereby increasing the rice grain yield. These results suggest that NEAs can optimize the morphophysiological characteristics of roots through phytohormone regulation to adapt to different nutrient conditions, thereby promoting N accumulation and yield formation in rice.

## Introduction

Rice (*Oryza sativa* L.) is one of the world’s most important crops, with rice the staple food for more than half of the world’s population (Hua et al., 2015; Zhang et al., 2019). Therefore, increasing rice yield can crucially contribute to global food security. Nitrogen (N) is the most essential nutrient in rice production, and current high yields of rice are associated with the application of large amounts of N fertilizer (Xu et al., 2012). The use of N fertilizer for rice production in China accounts for 37% of the global total (Li et al., 2014). Although N supply drives productivity, the N use efficiency (NUE) in the current rice production system is low despite high N fertilizer input, which is the main factor limiting rice yield (Peng et al., 2006). Current research focuses on ways to synergistically improve the NUE of rice while increasing rice yield.

The root system is an important organ for the absorption and transportation of N and the direct use of soil nutrients, and thus a key contributor to rice yield (Wu and Cheng, 2014; Meng et al., 2019). Previous studies have suggested that manipulating root morphological and physiological traits can improve N uptake and thereby increase crop yield and the efficiency of N fertilizers in agricultural systems (Mi et al., 2010; Ju et al., 2015; Xin et al., 2019). Root morphology, spatial distribution in soil, root tip cell status, root activity, and root-sourced phytohormones play a crucial role in root function (Zhang et al., 2009; Mi et al., 2010; Uga et al., 2013; Sun et al., 2016; Gu et al., 2018). Much attention has been paid to increasing grain yield by improving root distribution, structure, and function in modern rice cultivars. However, little is known about the effect of N supply on root morphological and physiological traits and their relationships with N acquisition and grain yield formation over the entire growth cycle.

There are five broad categories of recognized phytohormones: indole-3-acetic acid (IAA), gibberellins (GA), cytokinin (CTK), abscisic acid (ABA), and ethylene (ETH). In addition, jasmonic acid, salicylic acid, brassinosteroids, and polyamines also have phytohormone characteristics. ABA is synthesized in almost all plant cells that contain chloroplasts and other plastids (Taiz and Zeiger, 2002). A previous study suggested that ABA and CTK are synthesized mainly by the root system and transported to the aboveground parts via transport tissue to regulate plant growth and development (Kende and Zeevaart, 1997). In recent years, ABA, CTK, and other phytohormones, and their regulatory effects have been the focus of crop physiology research. With the development of molecular biology, important progress has been made in understanding the biosynthesis and metabolic pathways of phytohormones, and the function of phytohormones in plant growth and crop yield formation. The response of phytohormones to biotic and abiotic stress, the transmission of phytohormones signals, and the molecular mechanism of phytohormone regulation have also been reported on Chauffour et al. (2019), Altmann et al. (2020), and Blázquez et al. (2020). However, there are few studies on the relationship between root phytohormones and root morphophysiological traits under field cultivation conditions over the entire plant growth cycle.

In the present study, four rice cultivars were assessed to (1) determine the morphological and physiological traits of rice root systems grown under deficient, intermediate, and sufficient N conditions, (2) determine the major root morphological and physiological traits that contribute to higher total N content (TNC) and grain yield (GY) during the entire growth cycle of rice grown in the field, and (3) explore the relationship between phytohormones and root morphophysiological traits throughout the growth period. This study improves our understanding of the roles of phytohormones in the regulation of root morphology and physiological traits in N acquisition and grain yield formation. This information could be used for research focused on improving N acquisition and grain yield of rice.

## Materials and Methods

### Plant Materials and Site Description

**FIGURE 1:**
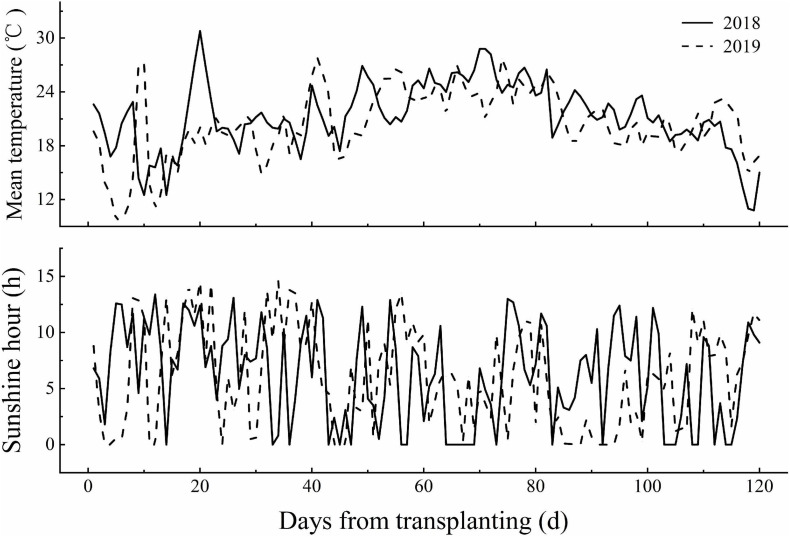
The mean temperature and sunshine hours during the growing season of rice in 2018 and 2019 at the experiment site.

Field experiments were conducted at the Harbin agriculture demonstration site in Heilongjiang Province, China (45°85′N, 126°46′E) during the rice growing season (April–October) in 2018 and 2019. The average air temperature and sunshine hours during the rice growing season in 2018 and 2019 are provided in Figure 1. Two N-efficient absorption cultivars (NEAs), Dongfu114 (DF114) and Longdao21 (LD21), and two N-inefficient absorption cultivars (NIAs), Longdao5 (LD5) and Longyang16 (LY16), were used. The four cultivars have similar growth periods (140–142 days). A crop rotation system was applied with continuous cropping of rice. The soil type was Mollisol. The physicochemical properties of composite topsoil samples (0–20 cm) were determined from samples collected through the five-point sampling method before partition, and the average values are shown in Table 1.

**TABLE 1:** The physicochemical property of composite topsoil samples (0–20 cm).

**Year**	**Organic matter**	**Total N content**	**Total P content**	**Total K content**	**Rapidly available N**	**Rapidly available P**	**Rapidly available K**	**Value of PH**
	**(g kg^–1^)**	**(g kg^–1^)**	**(g kg^–1^)**	**(g kg^–1^)**	**(mg kg^–1^)**	**(mg kg^–1^)**	**(mg kg^–1^)**	
2018	28.63	1.36	18.45	16.32	79.43	36.45	116.83	6.45
2019	27.91	1.32	17.63	16.34	78.36	35.84	117.6	6.42

### Experimental Design

The experiments were laid out in a complete randomized block design with three replicates. The N fertilizer rate was the main plot treatment, and the japonica cultivars formed the sub-plot treatments. The main plot size was 12 m in length and 9 m in width, with 30 cm row spacing and 13.3 cm terrace spacing, and two seedlings per hill. The main plots were separated at their perimeters by a polyvinyl chloride partition (0.14-cm thick, with 40.0 cm aboveground and 25 cm belowground) to prohibit the exchange of irrigation water and fertilizer between plots. Each plot has independent water inlet and outlet. The seeds were sown on April 14, 2018 and April 16, 2019. At the four-leaf heart stage, seedlings with similar growth were selected and transplanted on May 14, 2018 and May 16, 2019. The tests included three N application rate treatments: 60 (deficient N), 180 (intermediate N), and 240 (sufficient N) kg ha^–1^. N fertilizer was applied with basal, tillering, and panicle fertilizer at a ratio of 6:3:1 at the relevant growth stage. Phosphate fertilizer (P_2_O_5_) was applied once as a basal fertilizer at a rate of 90 kg ha^–1^. Potash (K_2_O) fertilizer was applied as a basal and panicle fertilizer at a ratio of 5:5, at a rate of 90 kg ha^–1^. N, P, and K fertilizers were used urea, Ca(H_2_PO_4_)_2_ and K_2_SO_4_, respectively. With the exception of the different N fertilizer application rates, the other cultivation requirements were identical for all plots in both years. Chemicals were used to control weeds, diseases, and insects to prevent yield loss. Avermectin is used as insecticide, butachlor, and pyrazosulfuron are used as herbicide, and tricyclazole is used as fungicide.

### Sampling and Measurements

Samples were collected between 9:00 am and 11:00 am on sunny days at the tillering, jointing, heading, filling (15 days after heading), and maturity stages. Plants were sampled in 18 (six replicates per cultivars × three replicates per treatment) hills with nine used to determine root morphological traits and shoots traits, and nine used to determine root physiological traits and phytohormone content (three replicates per cultivars are mixed into one sample × three replicates per treatment are used to determine hormone content). For each root sampling, a cube of soil (30.0 cm in length × 13.3 cm in width × 30.0 cm in depth) around each individual hill was removed using a sampling core. The cube contained approximately 95% of the total root biomass (Ju et al., 2015).

#### Sampling for Shoot Biomass and N Content

The shoot samples were fixed at 105°C for 30 min and then dried at 80°C until a constant weight was achieved. The N concentration in dried plant material was determined using an automatic Kjeldahl unit (FOSS-8400, Denmark), and the semi-micro Kjeldahl method (Cock et al., 1976). We used the accumulation of N in the aboveground parts at the maturity stage to represent the total N acquisition (TNC).

#### Root Morphological and Physiological Traits

Total root length (TRL) and root diameter (RD) were measured using WinRHIZO Pro 2013e software (Regent Instruments Inc., Quebec, Canada). To measure root dry weight (RDW), root samples were fixed at 105°C for 30 min and dried at 80°C to achieve a constant weight. The root oxidation activity (ROA) was determined using the method described by Ramasamy et al. (1997). The root total absorbing surface area (RTA) and the root active absorbing surface area (RAA) were determined using the methylthionine chloride dipping method (Xiao and Wang, 2005).

#### Root Phytohormones Content

Root phytohormones content determination was based on the method of Xin et al. (2019). The root samples were freeze dried with liquid N, after which 50 mg of the sample was placed in a 2 mL centrifuge tube, and 50 μL of internal standard solution added. Thereafter, 1 mL of acetonitrile aqueous solution (1% FA) was added and the sample was then mixed for 2 min. After extraction for 12 h at 4°C in the dark, the samples were centrifuged at 14,000 × *g* for 10 min. Thereafter 800 μL of the supernatant was freeze dried with liquid N, reconstituted with 100 μL of acetonitrile water (1:1, v/v), and centrifuged again at 14,000 × *g* for 10 min. The resultant supernatant was used for injection analysis.

The samples were separated using the Waters I-Class LC ultra-performance liquid chromatography system. During the mobile phase, the A solution was a 0.05% FA aqueous solution, and the B solution was 0.05% FA acetonitrile. The sample was placed in a 4°C autosampler, with a column temperature of 45°C, a flow rate of 400 μL/min, and an injection volume of 2 μL. The relevant liquid phase gradients were as follows: 0–10 min, liquid B which changed linearly from 2 to 98%; 10–10.1 min, liquid B which changed linearly from 98 to 2%; and 11.1–13 min, liquid B was maintained at 2%. One quality control sample was set for each interval of a certain number of experimental samples in the sample queue to test and evaluate the stability and repeatability of the system.

A 5500 QTRAP mass spectrometer (AB SCIEX, Boston, MA, United States) was used for mass spectrometry analysis in negative ion mode. The 5500 QTRAP ESI probe source conditions were as follows: source temperature 500°C, ion Source Gas1 (Gas1): 45, Ion Source Gas2 (Gas2): 45, Curtain gas (CUR): 30, and ionSapary Voltage Floating (ISVF) -4500 V. The mode detects the ion pair to be tested using positive polarity multiple reaction monitoring (VRM). Multiquant software was used to determine the chromatographic peak area and retention time. The content of plant hormones in the sample was calculated according to the standard curve.

#### GY and Its Components

GY was determined at maturity within a 1-m^2^ area for the three replicates in each plot, excluding border plants, and adjusted to a moisture content of 0.14 g H_2_O g^–1^ fresh weight. The plants in nine representative terraces in each plot were sampled to determine the yield components. Plant samples from each terrace were separated into straw and panicles. The panicle number from each terrace was recorded to determine the number of panicles per hectare. Filled and unfilled grains of the panicles were manually separated to measure the grain number per panicle and the seed setting rate. Randomly selected filled grains from each terrace were used to determine the 1,000-grain weight.

### Statistical Analysis

For experimental variables, one-way of variance (ANOVA) was applied to assess differences among treatments with SPSS 22.0 (Softonic International, Barcelona, Spain) software. Significant differences (*p* < 0.05) between treatments are indicated by different letters according to Fisher’s LSD. Graphs were drawn with edgeR software (Available online: http://www.r-project.org/) and Origin 2018 software (OriginLab, Northampton, MA, United States).

## Results

### Differences in GY and Its Components in Four Japonica Rice Cultivars Grown With Different Rates of N Availability

The GY increased with increasing N availability in all cultivars. The GY was significantly higher for NEAs than for NIAs under all N conditions. As shown in Table 2, effective panicles increased with increasing N rate in all cultivars. Compared with intermediate N conditions, the grain per panicle of DF114 and LY16 cultivars was significantly decreased under deficient N conditions, and the grain per panicle rate in all cultivars was significantly decreased under sufficient N conditions. Compared with the deficient and intermediate N conditions, the 1,000-grain weight of all cultivars was significantly decreased under sufficient N conditions. The seed setting rate decreased with increasing N rate in all cultivars, which was significantly higher for NEAs than for NIAs under intermediate and sufficient N conditions, implying that a higher GY for NEAs under intermediate and sufficient N conditions was mainly attributed to a higher seed setting rate. The analysis of variance showed that the yield and its components showed significant differences among cultivars, N treatments, and the interaction between variety and N treatment (Table 2). Similar results were obtained for root morphophysiological traits and phytohormone content (Supplementary Table 1). Variation in most traits (such as seed setting rate, 1,000-grain weight, and TNC) due to year and the interactions between year and cultivars, as well as year and N treatment, was not significant.

**TABLE 2:** Grain yield and yield components of japonica rice cultivars in the field experiment in 2018 and 2019.

**Year**	**Treatment**	**Cultivars**	**Grain yield (t ha^–1^)**	**Effective panicles (per m^–2^)**	**Grain per panicle**	**Seed setting rate (%)**	**1,000-grain weight (g)**
2018	Deficient N	DF114	6.64 Ac	269.88 Ac	117.00 Bb	91.92 Ba	23.43 Aa
		LD21	6.31 Bc	280.24 Ac	119.78 Bb	92.73 Aa	21.83 Ca
		LD5	5.51 Dc	275.29 Ac	103.90 Ca	92.97 Aa	22.63 Ba
		LY16	5.88 Cc	235.15 Bc	128.96 Ab	92.57 ABa	22.47 Ba
	Intermediate N	DF114	8.22 Ab	316.89 Cb	125.06 Ba	90.26 Ab	23.47 Aa
		LD21	7.98 Bb	367.86 Bb	121.77 Ba	88.77 Bb	21.73 Ca
		LD5	7.07 Cb	392.48 Ab	102.99 Ca	85.28 Db	21.60 Cb
		LY16	7.27 Cb	286.82 Db	138.92 Aa	86.75 Cb	22.23 Ba
	Sufficient N	DF114	8.96 Aa	403.80 Ca	115.89 Bb	88.57 Ac	22.17 Ab
		LD21	8.71 Ba	442.77 Ba	116.98 Bb	87.22 Bb	19.70 Cb
		LD5	8.07 Ca	487.70 Aa	98.16 Cb	83.06 Dc	20.77 Bc
		LY16	8.04 Ca	356.64 Da	129.22 Ab	84.91 Cc	21.23 Bb
2019	Deficient N	DF114	6.78 Ac	238.52 Dc	108.78 Cc	91.39 Ba	22.53 Bb
		LD21	6.71 Ac	256.28 Cc	121.62 Bb	92.59 Aa	21.83 Ca
		LD5	6.05 Bc	269.43 Bc	124.77 ABa	92.74 Aa	22.57 Ba
		LY16	6.26 Cc	281.14 Ac	129.56 Ac	92.98 Aa	23.70 Aa
	Intermediate N	DF114	8.50 Ab	339.48 Bb	126.82 Ba	90.54 Aa	23.57 Aa
		LD21	8.34 Ab	374.16 Ab	127.25 Ba	88.51 Bb	21.58 Cb
		LD5	7.36 Bb	380.14 Ab	109.09 Cb	87.02 Cb	21.44 Cb
		LY16	7.53 Bb	293.62 Cb	139.72 Aa	86.82 Cb	22.43 Ba
	Sufficient N	DF114	9.33 Aa	406.64 Ca	115.08 Cb	87.82 Ab	22.23 Ac
		LD21	9.10 Aa	460.90 Ba	120.21 Bb	86.39 Ac	19.63 Dc
		LD5	8.37 Ba	490.67 Aa	100.73 Dc	82.98 Bc	20.81 Cc
		LY16	8.49 Ba	367.76 Da	133.23 Ab	83.82 Bc	21.45 Bb
*F*-value	N		4688.42**	4222.15**	40.45**	825.87**	184.99**
	C		498.97**	701.49**	289.31**	83.05**	141.83**
	Y		265.05**	6.12**	20.15**	0.7	0.35
	N × C		5.60**	65.99**	4.29**	38.34**	7.75**
	N × Y		3.48*	6.79**	0.44	5.54**	0.1
	C × Y		0.26	4.37**	1.22	2	0.78
	N × C × Y		0.33	4.02**	0.71	1.28	0.15

*^∗^ and ^∗∗^*F* values significance at 0.05 and 0.01 probability levels, respectively. Different lowercase and uppercase letters represent significant differences among treatments and cultivars, respectively.*

### Differences in Shoot Dry Weight (SDW), RDW, and TNC

**FIGURE 2:**
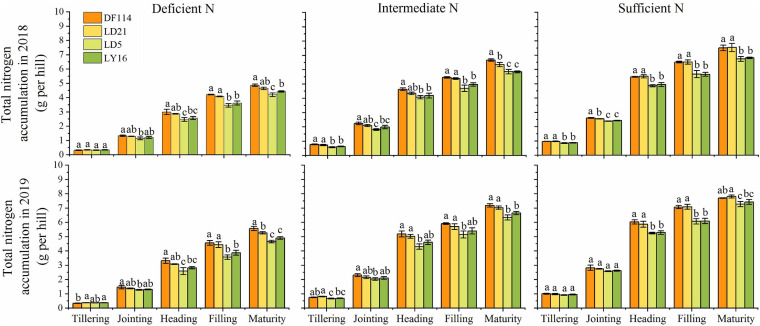
Total N content (TNC) of japonica rice cultivars from the tillering to maturity stage when grown with a deficient N, a intermediate and a sufficient N conditions in 2018, 2019. Each value is the mean (±SE) of three replicates. The different small letters above the column indicate significant difference among cultivars in the same N condition during the same growth stage at *P* < 0.05.

**FIGURE 3:**
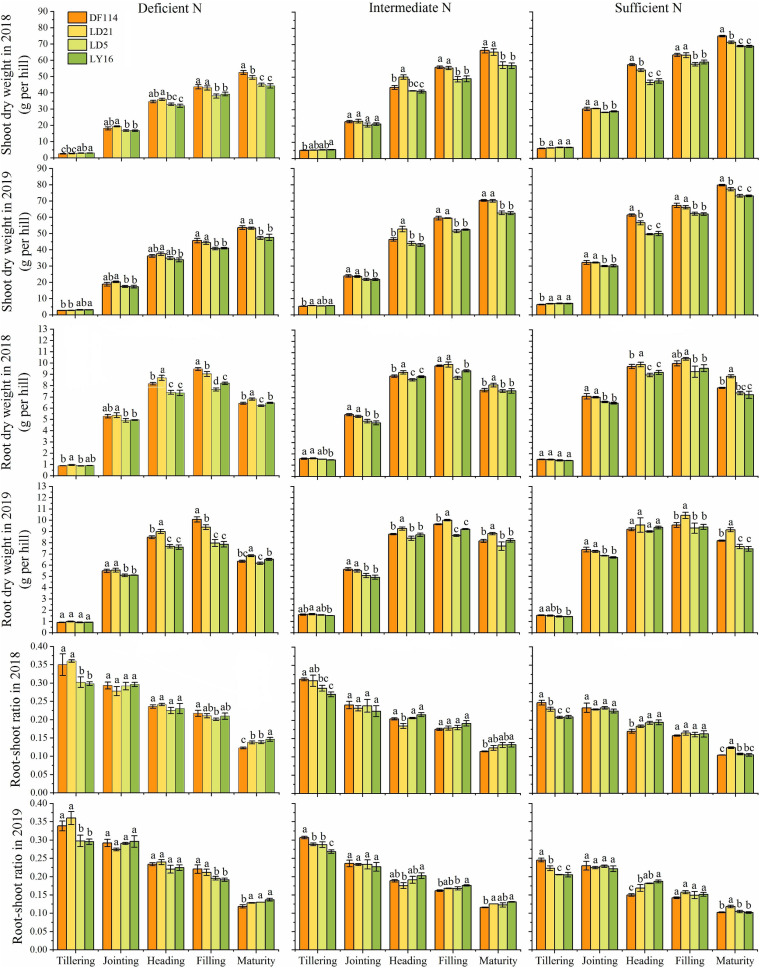
Shoot dry weight (SDW), root dry weight (RDW), and root-shoot ratio of japonica rice cultivars from the tillering to maturity stage when grown with a deficient N, a intermediate and a sufficient N conditions in 2018, 2019. Each value is the mean (±SE) of three replicates. The different small letters above the column indicate significant difference among cultivars in the same N condition during the same growth stage at *P* < 0.05.

The SDW and TNC of all cultivars increased gradually from the tillering to the maturity stage under all N conditions (Figures 2, 3). The SDW was significantly higher for NEAs than for NIAs from the jointing to the maturity stage under all N conditions. The TNC was significantly higher for the NEAs than for the NIAs at the heading to the maturity stage under all N conditions. The RDW first increased and then decreased from the tillering to the maturity stage, and all cultivars had the largest RDW in the filling stage under all N conditions. The RDW was significantly higher for NEAs than for NIAs from the jointing to the filling stage under deficient and intermediate N conditions, except for the heading stage in 2019 (Figure 3). The largest root-shoot ratio was observed at the tillering stage in all cultivars, and the root-shoot ratio was higher for the NEAs than for NIAs at the tillering stage under all N conditions (Figure 3). The results indicated that NEAs may promote root growth by regulating the root-shoot relationship in the early growth stage, which laid the foundation for N-efficient absorption.

### Differences in Root Morphological Traits

**FIGURE 4:**
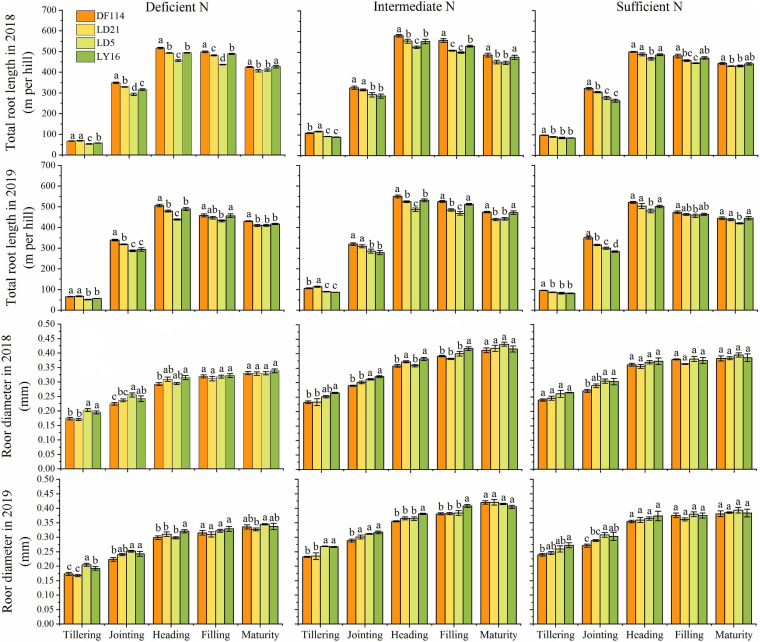
Total root length (TRL) and root diameter (RD) of japonica rice cultivars from the tillering to maturity stage when grown with a deficient N, a intermediate and a sufficient N conditions in 2018, 2019. Each value is the mean (±SE) of three replicates. The different small letters above the column indicate significant difference among cultivars in the same N condition during the same growth stage at *P* < 0.05.

The TRL of all cultivars increased rapidly from the tillering to the heading stage, and then decreased from the heading to the maturity stage under all N conditions (Figure 4). The TRL was significantly higher for NEAs than for NIAs from the tillering to the jointing stage under deficient and intermediate N conditions. The RD of all cultivars increased from the tillering to the maturity stage. The RD (except for the jointing stage under intermediate N conditions in 2019) was significantly lower for NEAs than for NIAs from the tillering to the jointing stage under deficient and intermediate N conditions, and it showed no significant difference between NEAs and NIAs from the heading to the maturity stage under all N conditions. Overall, NEAs may expand the distribution of roots in the growth environment by promoting root elongation in the early growth stage.

### Differences in Root Physiological Traits

**FIGURE 5:**
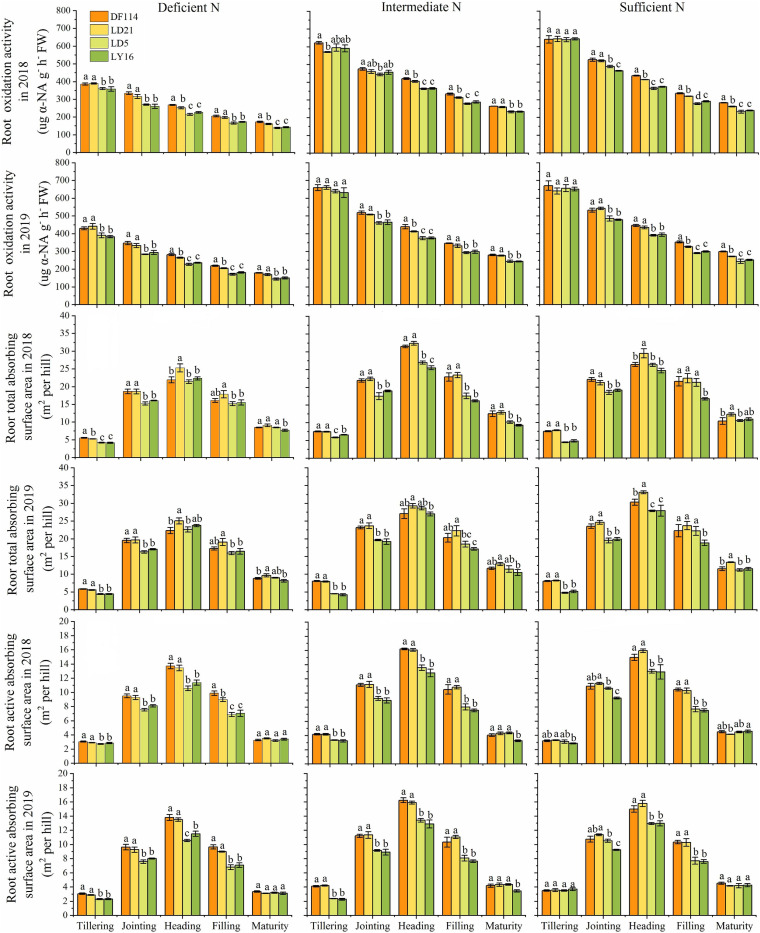
Root oxidation activity (ROA), root total absorbing surface area (RTA), and root active absorbing surface area (RAA) of japonica rice cultivars from the tillering to maturity stage when grown with a deficient N, a intermediate and a sufficient N conditions in 2018, 2019. Each value is the mean (±SE) of three replicates. The different small letters above the column indicate significant difference among cultivars in the same N condition during the same growth stage at *P* < 0.05.

The ROA of all cultivars decreased gradually from the tillering to the maturity stage under all N conditions, and the ROA was smaller when plants were grown under deficient N conditions than when grown under other N conditions at all growth stages (Figure 5). The RTA and RAA of all cultivars increased rapidly from the tillering to the heading stage, and then decreased from the heading to the maturity stage under all N conditions. The ROA (except for at the jointing stage under intermediate N conditions in 2018) and RAA were also significantly higher for the NEAs than for the NIAs from the jointing to the filling stage under all N conditions. The RTA was significantly higher for the NEAs than for the NIAs in the tillering to jointing stages under all N conditions. Interestingly, compared with other N conditions, the ROA and RAA of NEAs were significantly higher than those of NIAs at the tillering stage under deficient N conditions, which suggests that NEAs can regulate root physiological traits earlier to adapt to lower N stress more quickly.

### Correlations Between TNC, SDW, GY, and Root Morphological and Physiological Traits

**FIGURE 6:**
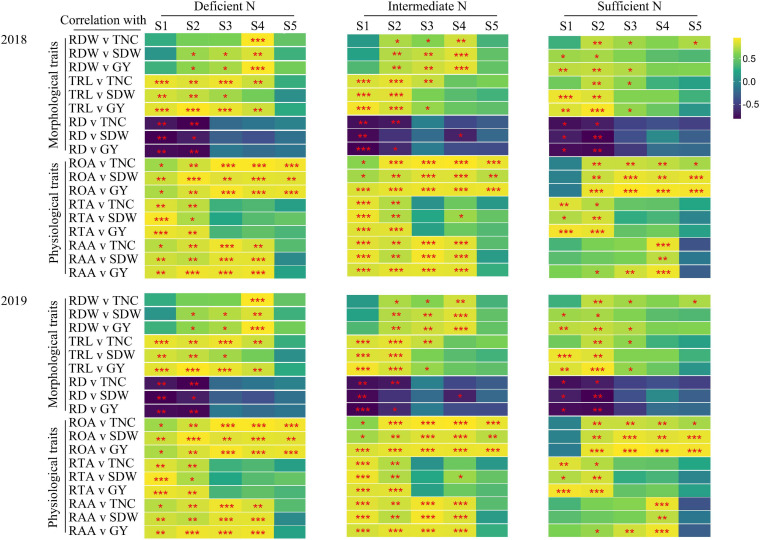
Pearson’s correlations between TNC (maturity stage), SDW (maturity stage) as well as grain yield (GY) and root morphological and physiological traits for 2018, 2019 in plants grown with a deficient N, a intermediate and a sufficient N conditions at different stage (S1-S5). S1, tillering stage; S2, jointing stage; S3, heading stage; S4, filling stage; S5, maturity stage. *P < 0.05, **P < 0.01, ***P < 0.001.

The correlations between TNC (at maturity stage), SDW, GY, and root morphological and physiological traits is shown in Figure 6. Under all N conditions, root morphological (except RD) and physiological traits at the main growth stages were strongly and positively correlated with TNC, SDW, and GY. The correlations between root morphological and physiological traits and TNC, SDW, and GY were different under all N conditions. Under deficient and intermediate N conditions, root morphological (except RDW) and physiological traits were significantly related to TNC, SDW, and GY at the tillering stage; root morphological and physiological traits were significantly related to TNC (except RDW), SDW (except RD), and GY at the jointing stage. Under sufficient N conditions, the ROA and RAA were not significantly related to TNC, SDW, and GY, and the significant level of correlation between the other traits and TNC, SDW, and GY were also lower than that under deficient and intermediate N conditions at the tillering stage, while the ROA and RAA were significantly related to TNC, SDW, and GY at the filling stage.

### Differences in Root Phytohormone Levels

**FIGURE 7:**
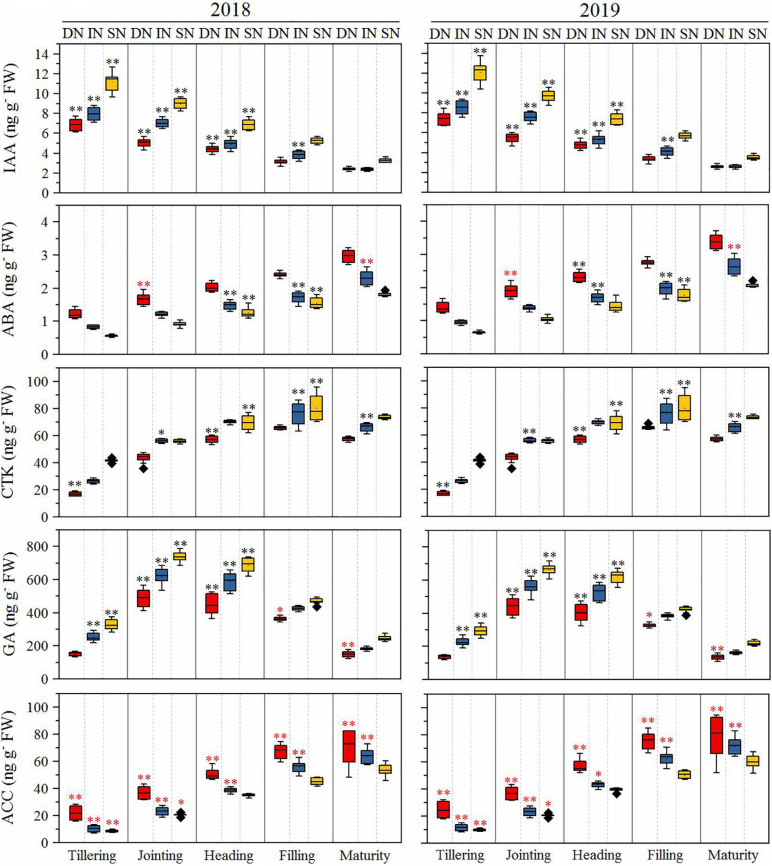
The IA, ABA, CTK, GA, and ACC of japonica rice cultivars from the tillering to maturity stage when grown with a deficient N, a intermediate and a sufficient N conditions in 2018, 2019. DN, Deficient N conditions; IN, Intermediate N conditions; SN, Sufficient N conditions. * and ** indicate that NEAs and NIAs are significantly different at the 0.05 and 0.01 levels, respectively; black *, ** means NEAs is higher than NIAs; red *, **means NEAs is lower than NIAs.

The root phytohormones are presented in Figure 7. The GA content of all cultivars increased rapidly from the tillering to the jointing stage, then decreased from the jointing to the maturity stage under all N conditions, and was significantly higher in NEAs than in NIAs from the tillering to the heading stage under all N conditions (deficient N conditions at tillering stage). The ABA content of all cultivars increased from the tillering to the maturity stage under all N conditions and was significantly higher in NEAs than in NIAs in the heading to the filling stage under intermediate and sufficient N conditions. The IAA content of all cultivars decreased from the tillering to the maturity stage under all N conditions and was significantly higher in NEAs than in NIAs from the tillering to the heading stage under all N conditions. The CTK content of all cultivars increased from the tillering to the maturity stage under all N conditions and was significantly higher in NEAs than in NIAs at the filling stage under intermediate and sufficient N conditions. The 1-aminocyclopropane-1-carboxylic acid (ACC) content of all cultivars increased from the tillering to the maturity stage under all N conditions and was significantly lower in NEAs than in NIAs from the tillering to the jointing stage under all N conditions and from the heading to the maturity stage under deficient and intermediate N conditions.

### Correlations Between Root Morphological and Physiological Traits and Root Phytohormones

**FIGURE 8:**
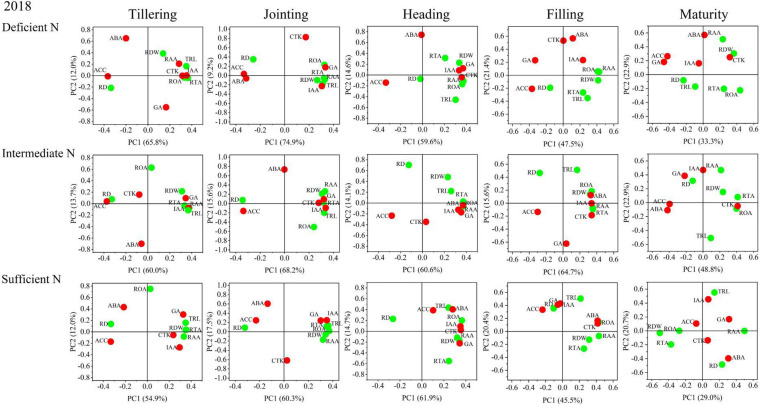
Principal component analysis (PCA) of phytohormones, root morphological and physiological traits determined on japonica rice cultivars when grown with a deficient N, a intermediate and a sufficient N conditions in 2018.

**FIGURE 9:**
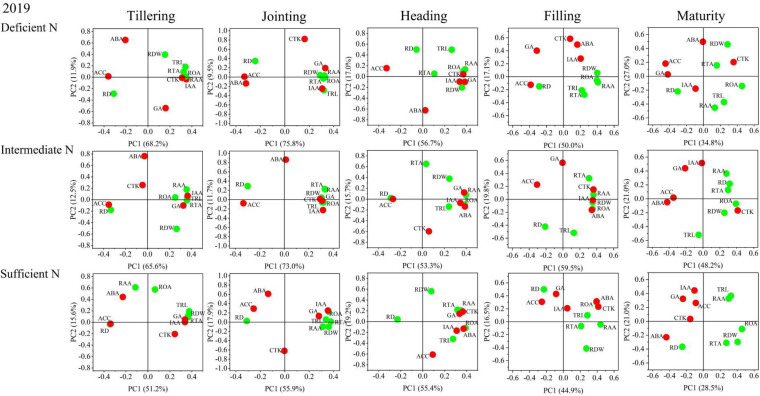
Principal component analysis (PCA) of phytohormones, root morphological and physiological traits determined on japonica rice cultivars when grown with a deficient N, a intermediate and a sufficient N conditions in 2019.

The results of the principal component analysis (PCA) are shown in Figures 8, 9 and indicated that the morphology and physiology of rice roots are closely related to phytohormones in the main growth stages. Under all N conditions, the ACC content was significantly related to RD, and the IAA content was significantly related to TRL, RTA, and RAA from the tillering to the jointing stage. Under intermediate and sufficient N conditions, the ABA and CTK was significantly related to ROA and RAA from the heading to the filling stage. These results showed that the IAA and ACC are closely related to the root morphological traits in the early growth stage under all N conditions, while ABA and CTK are closely related to the root physiological traits in the middle and late growth stages under higher N conditions.

### Correlations Between Seed Setting Rate and ROA at the Filling Stage

**FIGURE 10:**
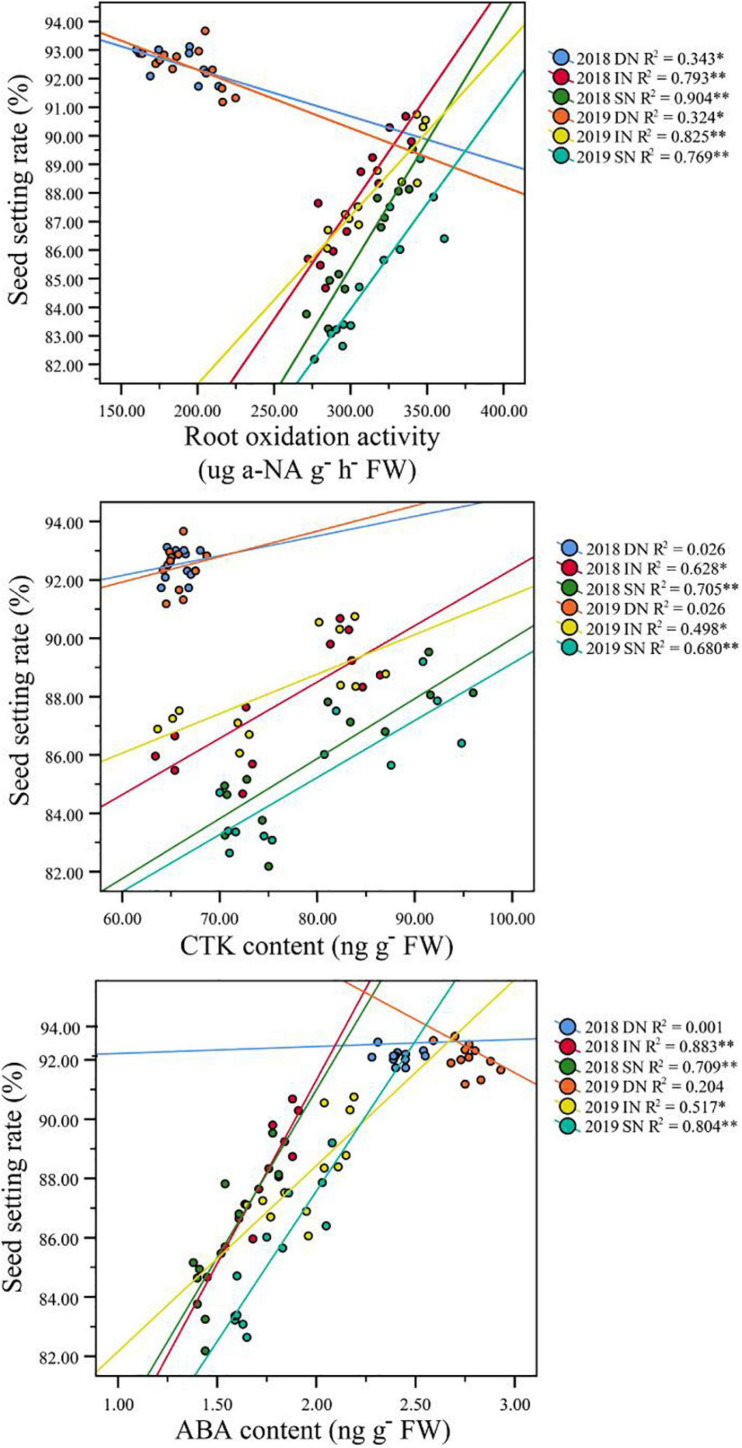
Pearson’s correlation between seed setting rate and ROA, CTK, and ABA content of japonica rice cultivars grown with a deficient N, a intermediate and a sufficient N conditions at filling stage. DN, Deficient N conditions; IN, intermediate N conditions; SN, Sufficient N conditions.

Correlation analysis results also showed that a higher GY in NEAs was mainly attributed to a higher seed setting rate under higher N conditions (Table 3). Under intermediate and sufficient N conditions, the difference in seed setting rate between NEAs and NIAs may be attributed, at least partially, to the difference in ROA during grain filling. Regression analysis showed that the mean ROA, CTK, and ABA contents during grain filling were significantly positively correlated with the seed setting rate under intermediate and sufficient N conditions (Figure 10).

**TABLE 3:** Correlations between grain yield and yield components of japonica rice cultivars for 2018, 2019 in plants grown with a deficient N, a intermediate and a sufficient N conditions.

**Year**	**Condition**	**Effective panicles (per m^–2^)**	**Grain per panicle**	**Seed setting rate (%)**	**1,000-grain weight (g)**
2018	Deficient N	0.44	0.19	-0.54	0.09
	Intermediate N	-0.01	0.14	0.90^∗∗^	0.46
	Sufficient N	-0.03	0.08	0.90^∗∗^	0.2
2019	Deficient N	0.36	0.3	-0.4	0.18
	Intermediate N	0.26	0.14	0.86^∗∗^	0.33
	Sufficient N	-0.1	0.08	0.93^∗∗^	0.11

*^∗∗^ indicate significant at the 0.01 probability levels.*

## Discussion

The NUE is a complex indicator, and according to the process of N absorption, assimilation, transportation, and distribution in plants, there are several definitions of NUE, mainly including N uptake efficiency, N utilization efficiency, N physiological use efficiency, agronomy efficiency of fertilizer N, N transport efficiency, apparent N recovery rate, and N remobilization efficiency (Xu et al., 2012). A number of reviews have summarized the broader aspects of NUE (Glass et al., 1992; Good et al., 2004; Fageria and Baligar, 2005; Hirel et al., 2007; Garnett et al., 2009; Robertson and Vitousek, 2009; Masclaux-Daubresse et al., 2010). In general, plants obtain N from soil N to produce biomass, which constitutes the NUE of plants. In the present study, N accumulation in the maturity stage under three N conditions was used as the evaluation index to analyze two NEAs and two NIAs, which were used to study the effect of N uptake on grain yield, in order to clarify the relationship between root morphophysiological traits and N uptake, and to understand relation between phytohormones and root morphophysiological traits.

### Relationship Between Plant N Uptake and Grain Yield

Previous studies on the relationship between rice yield and N accumulation found that rice has high N accumulation at the maturity stage under high-yield conditions (Du et al., 2007; Bingham et al., 2012). In the present study, the TNC and GY increased with the level of N application, and were significantly higher in NEAs than NIAs. Aboveground dry matter accumulation is the material basis for the formation of rice yield (Huang et al., 2019; Chen et al., 2020). Dry matter production of rice from heading to maturity stage accounts for approximately 90% of grain weight (Dordas, 2009), and the more dry matter accumulation from the heading to maturity stage, the higher the yield (Nagata et al., 2001; Jia et al., 2019; Deng et al., 2020). In the present study, we observed that NEAs have a greater TNC, which was closely associated with the greater SDW from the heading to maturity stage. Increasing the accumulation of dry matter and nitrogen at the late stage of spikelet differentiation could promote the formation of large spikes (Mae, 1997). Therefore, we concluded that an improved N uptake, as in larger biomass, contributes to a higher GY in NEAs.

### Relationship Between Root Morphophysiological Traits and N Uptake

Compared with NIAs, NEAs had evident advantages in RDW, TRL, ROA, RTA, and RAA at each growth stage, indicating that the improvement in N uptake capacity was accompanied by improvement in root morphology and physiological activity (Zhang et al., 2009; Fan et al., 2010; Ju et al., 2015; Xin et al., 2019). In addition, NEAs have a greater root to shoot ratio at the tillering stage because NEAs can mobilize more carbohydrates for root growth and use limited carbohydrates to activate root physiological functions, promote elongation growth (TRL), and reduce lateral growth (RD) at the early stages of growth, laying the foundation for beneficial root morphological and physiological characteristics in the mid- and late growth stage. Fan et al. (2010) showed that under two N supply levels, the ROA of N-efficient rice (Nanguang) was significantly higher than that of N-inefficiency rice (Elio), which is an important factor for its high N uptake. Ju et al. (2015) reported that N-efficient rice had larger root biomass and longer root length. Some studies have suggested that root and shoot growth inevitably compete for carbohydrate demand, and thus, roots with larger morphophysiological characteristics will consume more energy and carbohydrates and limit the growth of shoots (Samejima et al., 2004, 2005). In the present study, although NEAs had greater root growth and root vitality at the mid- and late stages, they also had greater accumulation of aboveground N and dry matter, and thus higher yields.

Root traits show great plasticity in the adaptive responses of rice to different rates of N supply (Zhang et al., 2009; Ju et al., 2015; Xin et al., 2019). In the present study, compared with NIAs, NEAs exhibited higher RL, root to shoot ratio, ROA, and RAA and smaller RD at the tillering stage under deficient N conditions. This indicated that NEAs adapted earlier and showed morphological and physiological adaptations to lower N stress. Many studies have indicated that in the late growth stage, rice root activity traits were positively correlated with yield (Fan et al., 2010; Ju et al., 2015; Zhang et al., 2019). Zhang et al. (2009) observed that the seed setting rate of rice was significantly positively correlated with the ROA in the mid- and late stages of grain filling. It is generally believed that N-efficient rice has larger roots and higher physiological activity, and the decline in root activity is slow in the late growth stage (Cassman et al., 2002; Fan et al., 2010). With the increase in N supply, the relationship between morphological traits in the middle and late stages and N uptake and yield formation gradually weakened, indicating that the physiological traits of roots were the main factors limiting yield in the middle and late stages under higher N conditions. These results indicated that NEAs can coordinate root morphology and physiology to obtain more N and produce higher yields under different N supply conditions.

### Relationship Between Phytohormones and Root Morphophysiological Traits

Phytohormones play an important role in synergistically regulating the growth and development of rice organs, nutrient absorption, carbon and N assimilation, transport and distribution as well as inducing defensive adaptation to stress (Xin et al., 2019). In terms of the relationship between phytohormones and N, studies have shown that CTK and IAA can regulate rice root morphogenesis and metabolite distribution (Albacete et al., 2014). CTK synthesis is regulated by N nutrition, which can inhibit rice root elongation and branch formation. CTK in the stems can enhance the expression of the nitrate transporter (NRT) and promote the distribution and transport of nitrates, while CTK in the roots inhibits NRT expression and reduces the absorption of nitrate by roots (Kiba et al., 2011). IAA has been shown to promote the formation of branched and adventitious roots of *Arabidopsis*, as well as the distribution of assimilation to the root system. ABA is involved in rice root growth and N acquisition (Peng et al., 2007), and GA and CTK can significantly slow wheat root senescence (Liu et al., 2013). In the present study, IAA, CTK, and GA levels increased and ABA and ACC levels decreased with an increase in the N supply level, which is consistent with previous studies (Krouk et al., 2011; Luo et al., 2015). The results of the PCA showed that IAA and ACC were closely related to the transverse and longitudinal growth of roots at the tillering and jointing stages, while CTK and ABA were closely related to the physiological indices of roots at the heading and filling stage, which further demonstrated the fact that hormone content difference is the physiological basis of N-efficient absorption varieties.

We conclude that NEAs regulate TRL and RD through a higher IAA and ACC contents at the early stages of rice growth, laying a morphological basis for its high N uptake efficiency. Previous studies have indicated that CTK is an important index of root physiology, which is closely related to root vitality traits and the filling rate at the filling stage (Saini and Westgate, 1999; Yang et al., 2003; Javid et al., 2011). Higher root vitality plays an important role in maintaining the growth of roots and aboveground parts and ion absorption (Zhang et al., 2009; Ju et al., 2015). ABA is generally recognized as an inhibitory plant hormone. However, recent studies have found that ABA has a positive regulatory effect on the grain filling of crops, such as wheat and rice (Kato, 2004; Peng et al., 2006; Dong et al., 2014), which is consistent with the results of the present study. We observed that the ROA and root ABA and CTK content during grain filling were significantly correlated with the seed setting rate under higher N conditions, implying that improving the content of ABA and CTK in rice roots during the filling stage can increase root activity, thereby increasing the yield potential of rice. These results indicate that phytohormones content is closely related to root morphophysiological characteristics, and can respond to nitrogen availability to regulate root morphophysiological characteristics at different growth stages to maintain normal growth.

## Conclusion

Compared with NIAs, NEAs showed a stronger tolerance to low N stress, and a higher yield potential under higher N supply conditions. Under lower N conditions, compared with NIAs, NEAs showed greater TRL, ROA, and RAA and smaller RD owing to higher IAA and CTK, and lower ACC content in the early growth stages, to respond to low N stress faster, laying a morphophysiological basis for its high N uptake capacity in the mid- and late growth stages. Under higher N conditions, NEAs had higher ROA and RAA for N uptake and yield formation owing to higher ABA and CTK content in the mid- and late growth stages, which improved the seed setting rate, thereby increasing the grain yield of rice. These results indicated that phytohormones play an important role in the regulation of root morphophysiological characteristics and N uptake. Further research is needed to understand the mechanism of exogenous phytohormones (e.g., application concentration, ratio, and period) in the regulation of root growth and N uptake and to improve the N uptake and yield in rice.

## Data Availability Statement

The original contributions presented in the study are included in the article/Supplementary Material, further inquiries can be directed to the corresponding author.

## Author Contributions

DZ and WX designed the study and provided experimental materials. WX analyzed the results, prepared the figures and tables, and wrote the manuscript. All authors discussed the results, commented on the manuscript, read, and approved the final manuscript.

## Conflict of Interest

The authors declare that the research was conducted in the absence of any commercial or financial relationships that could be construed as a potential conflict of interest.

## Publisher’s Note

All claims expressed in this article are solely those of the authors and do not necessarily represent those of their affiliated organizations, or those of the publisher, the editors and the reviewers. Any product that may be evaluated in this article, or claim that may be made by its manufacturer, is not guaranteed or endorsed by the publisher.

## Supplementary Material

Supplementary Table 1 Analysis-of-variance of *F*-values of grain yield, yield components, Shoot dry weight (SDW), root dry weight (RDW) and root-shoot ratio, Total N content (TNC), Total root length (TRL), root diameter (RD), Root oxidation activity (ROA), root total absorbing surface area (RTA), root active absorbing surface area (RAA), indole-3-acetic acid (IAA), gibberellins (GA), cytokinin (CTK), abscisic acid (ABA) and 1-aminocyclopropane-1-carboxylic acid (ACC) of rice between/among years, varieties, and N treatments. ^∗^ and ^∗∗^ *F* values significance at 0.05 and 0.01 probability levels, respectively.
